# Development, Validation, and Evaluation of a Nurse‐Led Perioperative Staged Exercise Management Program for Arteriovenous Fistula: A Sequential Multiphase Study

**DOI:** 10.1155/jonm/1430814

**Published:** 2026-08-09

**Authors:** Yang Tang, Mengya Wang, Mengyao Liu, Pinli Lin, Xu Deng, Jialu Xu, Mei Ling, Yao Fang, Yuan Zhang, Yu Gao, Fang Tang, Yucheng Cao, Lili Deng

**Affiliations:** ^1^ School of Nursing, Guangzhou University of Chinese Medicine, Guangzhou Guangdong, 510006, China, gzucm.edu.cn; ^2^ School of Nursing, Hunan University of Chinese Medicine, Changsha Hunan, 410208, China, hnctcm.edu.cn; ^3^ School of Nursing, Guangdong Pharmaceutical University, Guangzhou Guangdong, 510310, China, gdpu.edu.cn; ^4^ Qionghai Institute of Nursing Excellence, Qionghai Hainan, 571434, China; ^5^ The Second Affiliated Hospital of Guangzhou University of Chinese Medicine, Guangzhou Guangdong, 510120, China, gzucm.edu.cn

**Keywords:** arteriovenous fistula, exercise adherence, perioperative nursing, self-management, vascular access nursing

## Abstract

**Objective:**

Arteriovenous fistula (AVF) is the preferred vascular access for hemodialysis, yet postoperative exercise recommendations are often inconsistent and support after discharge is limited. This study aimed to develop, validate, and preliminarily evaluate a nurse‐led perioperative staged exercise management program after AVF creation.

**Methods:**

This sequential multiphase study included a structured evidence review, Delphi expert consultation, pilot testing, and a single‐center prospective controlled evaluation. Adults undergoing forearm radiocephalic AVF creation were enrolled from July 2024 to February 2025 and allocated by temporal phase to usual care or intervention. The intervention included a staged exercise program, standardized inpatient instruction, discharge‐day confirmation, WeChat milestone reminders, and follow‐up checks. Ultrasound outcomes were assessed before surgery and at 8 weeks; maturation outcomes and process indicators were recorded. TREND guided reporting of the nonrandomized evaluation.

**Results:**

Two Delphi rounds achieved high authority and consensus (Cr > 0.90; *W* = 0.271, *p* < 0.001). Eighty‐two patients were enrolled, 41 per group, and all completed follow‐up. In the intervention group, completion of standardized exercise instruction reached 100.0%, mean instruction time was 12.4 min, and 82.9% of patients passed discharge‐day confirmation at the first check. Home exercise adherence increased over time. At 8 weeks, higher brachial artery blood flow (*p* < 0.001), larger brachial artery diameter (*p* = 0.007), and larger radial artery diameter (*p* = 0.039) were observed in the intervention group. The intervention group was also associated with higher 8‐week maturation (95.1% vs. 75.6%, *p* = 0.012), higher 12‐week maturation (100.0% vs. 82.9%, *p* = 0.012), and shorter time to maturation (*p* < 0.001), with no serious exercise‐related adverse events through 12 weeks.

**Conclusions:**

The nurse‐led perioperative staged exercise management program was feasible in ward practice and was associated with more favorable ultrasound and maturation‐related outcomes after AVF creation. Multicenter studies with stronger designs are needed to confirm generalizability and long‐term value.

**Implication for Nursing Management:**

High‐quality nursing management is crucial to patient recovery. In AVF care, postoperative support should not be reduced to one‐time education, but incorporated into a structured and continuous nursing management process to strengthen care transition, behavioral maintenance, and recovery support. This managerial implication may also be relevant to other clinical contexts requiring sustained postoperative management.

**Trial Registration:** International Traditional Medicine Clinical Trial Registry: ITMCTR2024000038

## 1. Introduction

Chronic kidney disease (CKD) has become a major global public health problem, and its burden continues to rise. Worldwide, CKD ranked as the 12th leading cause of disability‐adjusted life years in 2023 and has been projected to become one of the five leading causes of death globally by 2040 [1, 2]. When CKD progresses to end‐stage renal disease (ESRD), many patients require long‐term hemodialysis in addition to kidney transplantation and peritoneal dialysis [3]. Vascular access management therefore becomes a central part of dialysis preparation, and arteriovenous fistula (AVF) is widely regarded as the preferred vascular access because of its safety, durability, and ease of cannulation [4]. A study covering 527 hemodialysis centers in China reported that AVF use reached 80.9% [5]. Whether an AVF becomes a stable and usable access within the expected time depends on both the speed and quality of maturation. However, delayed or failed AVF maturation is common. Poor maturation prolongs the preparation period for dialysis and increases dependence on temporary central venous catheters, thereby raising the risks of infection, thrombosis, reoperation, or reintervention, as well as the burden on hospitalization, follow‐up, and nursing resources [6, 7]. Promoting perioperative AVF functional establishment, shortening the time to maturation, and improving clinical usability therefore remain important priorities in vascular access nursing [8].

Current strategies to promote AVF maturation focus largely on interventional procedures or pharmacologic approaches, but these strategies are influenced by individual variation and local resource availability [6]. By contrast, postoperative upper limb and hand exercises are commonly used nonpharmacologic interventions that are low cost, feasible, and easy to implement [9, 10]. Previous studies suggest that handgrip training, forearm resistance and rhythmic exercises, local heat therapy, and postural management may support fistula maturation by improving blood flow within the fistula, enhancing shear stress, and promoting venous dilation and remodeling [11, 12]. However, existing evidence remains inconsistent, with variations in exercise frequency, intensity, duration, rhythm, monitoring approaches, outcome selection, and timing of imaging or hemodynamic assessment. In real‐world nursing pathways, these interventions are often reduced to general advice such as staying active or exercising as tolerated, without a reproducible prescription or a complete implementation pathway. Key implementation questions often remain unanswered, including how exercises should be combined and presented, when they should be advanced or interrupted, how discomfort should be handled, and how reminders and follow‐up should be delivered after discharge. As a result, exercise intensity and consistency can vary markedly, and even potentially useful interventions may fail to become sustainable nursing practice. Recent studies have also shown that educational or behavioral interventions for long‐term vascular access self‐management in hemodialysis patients remain limited, and current evidence is still insufficient to show clearly how structured support may improve vascular‐access‐related behaviors. At the same time, barriers and facilitators related to self‐care and disease management among dialysis patients are highly individualized, suggesting that a continuous care pathway built around patients’ understanding, support, and participation may promote behavior better than one‐time education [13, 14]. In addition, the development of practice standards for patient education in nurse‐led clinics suggests that educational activities that lack clear structure, process, and outcome standards are difficult to implement consistently and difficult to evaluate, whereas standards‐based patient education is more likely to guide clinical practice and improve educational quality [15].

In response to these evidence gaps and practical challenges, this nurse‐led study conceptualized upper limb and hand exercise after AVF creation as a health behavior that must be sustained after discharge. The potential effects of postoperative exercise may depend first on whether patients are advised to exercise, and further on whether they can perform the exercises correctly, receive adequate external support, and sustain the behavior over time. To identify and embed the key conditions influencing behavior attainment, the capability, opportunity, motivation, and behavior (COM‐B) model was adopted as the theoretical framework for intervention development [16]. Therefore, this sequential multiphase study aimed to develop a standardized perioperative staged exercise management program, validate it through expert consensus, and preliminarily evaluate its feasibility and association with AVF maturation‐related outcomes using a single‐center prospective controlled design. By integrating exercise prescription, standardized instruction, postdischarge support, and follow‐up assessment into a continuous nurse‐led management pathway, this study sought to assess the implementability of the intervention in ward and postdischarge settings and to provide necessary implementation‐level evidence for interpreting differences in maturation outcomes.

## 2. Materials and Methods

### 2.1. Study Design

**FIGURE 1:**
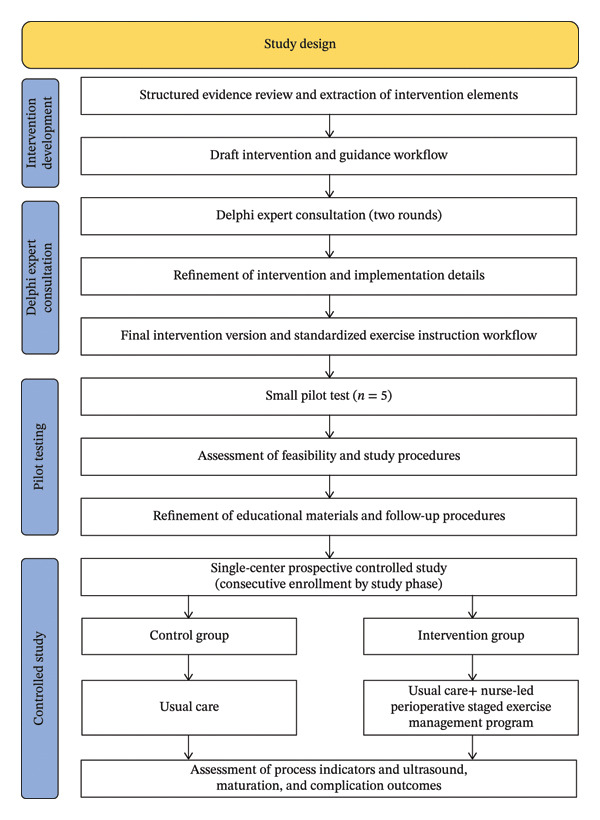
Study design and overall workflow of the nurse‐led perioperative staged exercise management program.

This study used a sequential, multiphase design consisting of four stages: a structured evidence review and extraction of intervention elements, two rounds of Delphi expert consultation, pilot testing, and a single‐center prospective controlled study. First, a draft intervention and guidance workflow were developed through a structured evidence review and extraction of key elements, and the intervention components and implementation procedures were organized according to the COM‐B framework. Within this framework, the intervention components were mapped to the behavioral conditions required for sustained postdischarge exercise performance. Capability‐oriented components focused on patients’ understanding and correct performance of AVF‐related exercise and self‐care requirements [17]. Opportunity‐oriented components provided reminder‐supported progression and follow‐up support, while motivation‐oriented components addressed uncertainty about pain, bleeding, traction injury, changes in AVF thrill, and safe exercise progression [18, 19]. A multidisciplinary expert panel then completed two rounds of Delphi consultation to refine the intervention content and exercise dose, stage‐specific progression milestones, the content covered in confirmation of key points and the principles for temporary interruption, the follow‐up process, the WeChat milestone reminder process, and the selection and timing of outcomes. This process produced a final intervention version and a standardized exercise instruction workflow. Before the formal clinical study, a small pilot test was conducted to examine the recruitment process, the operability of data collection forms, the reachability of follow‐up time points, and the completeness of safety‐event recording, and to refine the wording of structured educational materials and the scripts used in WeChat reminders. Finally, a single‐center prospective controlled study was conducted with consecutive enrollment by study phase to reduce contamination caused by the spread of health information and imitation of behaviors within the same ward. Differences in primary and secondary outcomes and complication outcomes were compared between groups. Process indicators, including implementation resource use and home exercise adherence, were also recorded to provide supplementary evidence of implementation feasibility in routine clinical wards and to support interpretation of clinical outcomes. The study design is shown in Figure 1.

### 2.2. Structured Evidence Review and Extraction of Intervention Elements

The exercise management program was developed by integrating evidence from a structured evidence review with clinical practice needs, and the intervention elements and implementation strategies were organized using the COM‐B model. The review focused on identifying upper limb and hand exercise interventions used to support AVF maturation after AVF creation and extracting intervention elements that could be translated into a perioperative staged exercise management program. Search strategies were constructed around three concept blocks, including the target population, the research topic, and the intervention. The target population was represented by “hemodialysis.” The research topic was represented by “arteriovenous fistula” and “AVF.” The intervention was represented by “exercise,” “exercise therapy,” “isometric exercise,” “training,” “upper limb exercise,” and “arm exercise.” Synonymous terms within each concept block were combined using OR, and the three concept blocks were combined using AND. Search strategies were adapted according to the indexing systems and search rules of each database. Chinese and English databases were searched, including PubMed, EBSCO, the Cochrane Library, Web of Science, Embase, China National Knowledge Infrastructure, Wanfang, VIP, and the Chinese Biomedical Literature Database. Search terms mainly included “hemodialysis,” “arteriovenous fistula,” “AVF,” “exercise,” “exercise therapy,” “isometric exercise,” “training,” “upper limb exercise,” and “arm exercise.” Detailed search strategies for each database are provided in Table S1. Searches covered the period from database inception to December 31, 2023.

Searches were conducted by one researcher according to the predefined strategies and checked by a second researcher. All retrieved records were imported into Zotero 7.0 for reference management. Duplicate records were removed through automatic deduplication combined with manual verification, and a traceable screening log was established. Literature screening was independently conducted by two researchers with nursing backgrounds, including title and abstract screening followed by full‐text screening. Disagreements were first discussed by the two researchers, with a third researcher consulted when necessary. The literature screening process is shown in Figure S1.

Data extraction was independently performed by two researchers and cross‐checked to ensure completeness and consistency. Based on the included studies, the research team conducted a structured analysis consisting of extraction of key intervention elements, screening for clinical feasibility, and development of a preliminary draft. When discrepancies occurred during data extraction or structured analysis, the two researchers returned to the original articles and reviewed the relevant context before reaching consensus through discussion. If consensus could not be reached, a third researcher was consulted.

Intervention schemes from each included randomized trial were extracted and categorized item by item into a matrix covering movement content and format, timing of initiation and duration, dose definition including repetitions, sets, duration, and frequency, staging or progression principles, interruption or contraindications and safety monitoring, educational and follow‐up approaches, adherence support and recording methods, and major outcomes (Table S2). This element matrix was then screened and reorganized in light of the ward‐based postoperative management pathway for AVF in our hospital and the feasibility of after‐discharge follow‐up. Movement modules that could be advanced across recovery stages without requiring expensive equipment or complex monitoring were retained. Dose parameters that could be expressed through time, movement combination, frequency, and total duration per session were retained. Progression milestones that could be triggered through WeChat reminders and aligned with follow‐up checks were identified. Safety prompts that could be implemented through patient self‐observation and complication follow‐up were retained, and a low‐burden self‐report recall method was selected for adherence assessment to maximize real‐world feasibility. These elements were simultaneously mapped onto the three COM‐B dimensions to ensure that the intervention content, instruction workflow, and follow‐up support formed a closed loop across capability, opportunity, and motivation.

### 2.3. Delphi Expert Consultation

#### 2.3.1. Expert Panel and Eligibility Criteria

Experts for the Delphi consultation were recruited from relevant specialties at Guangdong Provincial Hospital of Traditional Chinese Medicine. Two rounds of Delphi consultation were conducted. In Round 1, 15 experts participated, including 5 professors or chief nurses, 7 associate professors or associate chief nurses, and 3 attending physicians or nurse specialists. Their professional experience ranged from 10 to 30 years, with 8 experts having 10 to 20 years of experience and 7 having 20 to 30 years. The professional fields represented were nephrology (*n* = 6), hemodialysis care (*n* = 5), vascular surgery (*n* = 3), and rehabilitation medicine (*n* = 1), including 10 clinical experts and 5 nursing experts. In Round 2, 14 questionnaires were returned, yielding a valid response rate of 93.3%. One expert did not continue because of unexpected clinical commitments. Detailed characteristics of the experts in the two rounds are presented in Table S3.

#### 2.3.2. Questionnaire Content, Implementation, and Item Selection

Based on the draft intervention, a Delphi questionnaire entitled “Nursing program for staged hand exercise after AVF surgery” was developed. It included expert characteristics, importance ratings and revision suggestions for program items, and a self‐rated expert authority form. Item importance was rated on a 5‐point Likert scale from 1 = very unimportant to 5 = very important, and open‐ended fields were provided for comments and suggestions. Two rounds of consultation were conducted between February and March 2024. Fifteen experts completed Round 1, and 14 experts participated in Round 2. Items were screened using a mean importance score of at least 4.0, a coefficient of variation no greater than 0.25, and a consensus rate of at least 75%, together with group discussion to determine whether items should be retained, modified, or removed. The two rounds focused on whether stage‐specific milestones and movement progression matched the postoperative recovery trajectory; whether the total duration per session and daily frequency matched patient tolerance; whether the wording of milestones was suitable for triggering WeChat reminders and follow‐up checks after discharge; whether interruption principles and recommendations for seeking medical care were clear and practical; and whether the timing of outcome assessment corresponded to the maturation process. After the two rounds, a final intervention version and its standardized exercise instruction and reminder‐follow‐up process were established.

### 2.4. Pilot Testing

After the final intervention version had been completed, pilot testing was conducted to examine the feasibility of recruitment and intervention delivery, form completion, follow‐up procedures, and data collection. In April 2024, five patients with ESRD undergoing AVF creation were consecutively enrolled in an 8‐week pilot study. The pilot focused on the smoothness of the inpatient standardized exercise instruction workflow, patients’ understanding of the logic of staged progression, the reach of WeChat reminders, the attainability of follow‐up time points, and the completeness of recording adverse events and complications. It also standardized the completion rules and data structure of process indicators, including time required for standardized exercise instruction, result of confirmation of key points, and number of completed sessions in the past 7 days. The inclusion and exclusion criteria for the pilot were identical to those of the formal clinical study.

### 2.5. Controlled Clinical Study

#### 2.5.1. Participants and Recruitment

This single‐center prospective controlled study was conducted in the Department of Nephrology, Second Affiliated Hospital of Guangzhou University of Chinese Medicine, from July 2024 to May 2025. Because health education in inpatient wards may give rise to contamination through information exchange and imitation of behaviors, patients were enrolled and allocated by study phase. This approach was used to reduce within‐ward contamination, but it may have introduced temporal bias because the two groups were recruited during different periods. Consecutive eligible patients recruited from July to October 2024 were assigned to the control group. After control group enrollment was completed, consecutive eligible patients recruited from November 2024 to February 2025 were assigned to the intervention group. All participants provided written informed consent before enrollment. The reporting of this nonrandomized controlled evaluation followed the Transparent Reporting of Evaluations with Nonrandomized Designs (TREND) guideline.

Inclusion criteria were as follows: diagnosis of chronic renal failure with an estimated glomerular filtration rate below 25 mL/(min·1.73 m^2^) and an expectation that hemodialysis would be required within 3 to 6 months [20]; age 18 to 85 years; a forearm cephalic vein that was patent, soft, and compressible, with a tourniquet enhanced diameter of at least 2.0 mm; a radial artery (RA) with adequate flow, no marked calcification, and a diameter of at least 1.5 mm; planned creation of a radiocephalic AVF in the nondominant forearm using an end‐to‐side venous to arterial anastomosis; and willingness to participate. Exclusion criteria were as follows: musculoskeletal disorders such as myopathy or rheumatoid arthritis; central or peripheral neurologic disease such as stroke or peripheral neuropathy; severe comorbid conditions such as heart failure, malignancy, or severe infection; and psychiatric disease or cognitive impairment.

#### 2.5.2. Sample Size Estimation

The sample size was calculated using brachial artery (BA) blood flow at 8 weeks after surgery as the primary continuous outcome. According to a previous study [21], BA flow in the control group was 673.3 ± 172.2 mL/min, and a between‐group increase of 120 mL/min was expected after the intervention. Assuming a power of 1 − *β* = 0.80 and a two‐sided *α* of 0.05, PASS 15.0 indicated that 34 participants would be required per group for a comparison of two independent means. Allowing for an anticipated 10% attrition rate, at least 38 patients were required in each group. In the present study, 82 patients were ultimately included, with 41 in each group.

The sample size formula was as follows:

(1) n1=n2=Z12−α/+Z1−β2×σ2δ2×1−γ,

where *n*_1_, *n*_2_: required sample sizes for the control group and the intervention group; *Z*_1−*α*/2_: critical value of the standard normal distribution corresponding to the significance level *α*; *Z*_1−*β*_: critical value of the standard normal distribution corresponding to the statistical power 1 − *β*; *σ*: population standard deviation; *δ*: expected difference in group means to be detected; and *γ*: anticipated loss to follow‐up rate.

### 2.6. Interventions

#### 2.6.1. Control Group (Usual Care)

The control group received usual care delivered by the nephrology ward nursing team according to the department’s routine perioperative AVF nursing practice and documented in routine nursing records. Usual care included postoperative local observation and instruction on access self‐checks, such as observing skin color, temperature, and swelling; identifying bleeding or exudation; listening for vascular bruit; and palpating for thrill, together with protection of the access arm by avoiding blood pressure measurement, blood sampling, intravenous infusion, constrictive accessories, compression, and weight bearing on the operated side, psychological care, and general exercise instruction. General exercise instruction included elevation of the affected limb after surgery to promote venous return and prevent swelling, and ball‐squeezing exercise starting 2 weeks after surgery. The control group did not receive staged progression reminders or WeChat milestone reminders.

#### 2.6.2. Intervention Group

In addition to usual care, the intervention group received a staged hand exercise program. The program followed the postoperative recovery trajectory, progressing from postural management and gentle fingertip activity to wrist exercises, fist clenching, and ball squeezing, with WeChat milestone reminders used after discharge to support progression. Exercise frequency was set at twice daily, once in the morning and once in the evening, with an interval of at least 8 h between sessions. Total duration per session was generally limited to 15 to 20 min. The program emphasized a stable rhythm and patient tolerance, avoided breath‐holding and the Valsalva maneuver, and followed the breathing principle of exhaling on exertion and inhaling during relaxation.

**FIGURE 2:**
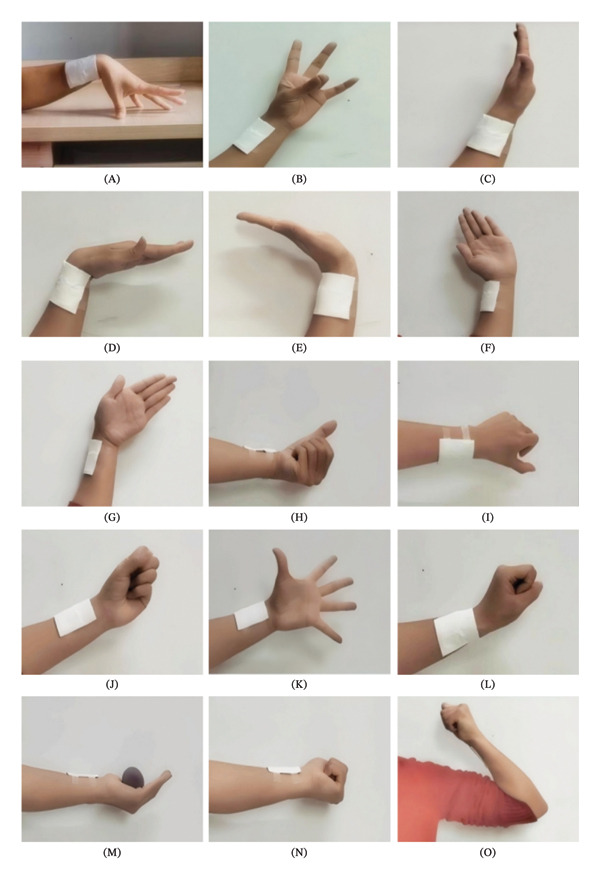
Exercise modules. Note: A = fingertip exercise; B = opposition exercise; C–E = wrist exercises in neutral position, palmar flexion and dorsiflexion; F–I = radial deviation, ulnar deviation, pronation and supination; J and K = fist‐clenching exercise; L = suspended wrist fist clenching; M and N = ball‐squeezing exercise; O = elbow flexion exercise.

The staged exercise program was organized into five postoperative stages according to recovery time and exercise tolerance. During the first 12 to 24 h after surgery, the focus was postural management and early gentle fingertip activity. On postoperative Day 2, fingertip and opposition exercises were introduced. From postoperative Days 3 to 6, wrist exercises and fist‐clenching exercises were added. From postoperative Days 7 to 14, ball squeezing and elbow flexion ball squeezing were introduced. From postoperative Day 14 to Week 8, patients entered the consolidation and progression phase. The stage timing, objectives, and core components of the staged hand exercise program are summarized in Table 1. Detailed operational procedures, including body position, movement sequence, movement duration, holding time, progression adjustment, interruption principles, and safety precautions, are provided in Supporting Table S4. The complete movement set is shown in Figure 2.

**TABLE 1:** Summary of the perioperative staged hand exercise program.

Stage	Timing	Stage objective	Core components
Stage 1	First 12–24 h after surgery	Support early recovery and prevent swelling or stiffness	Operated‐limb elevation, neutral wrist positioning, access observation, and gentle fingertip activity
Stage 2	Postoperative Day 2	Initiate low‐intensity hand activity	Fingertip tapping and thumb‐to‐finger opposition exercises
Stage 3	Postoperative Days 3–6	Add wrist mobility and fist‐clenching training	Wrist exercise and fist clenching, with suspended wrist fist clenching if tolerated
Stage 4	Postoperative Days 7–14	Introduce resistance‐based hand exercise	Soft elastic ball squeezing and elbow flexion ball squeezing
Stage 5	Postoperative Day 14 to Week 8	Consolidate exercise performance and support progressive training	Warm‐up, core exercise modules, relaxation, and staged progression according to tolerance

Safety monitoring and interruption principles remained the same throughout the program. Patients were advised to palpate the thrill over the anastomosis before and after exercise and compare it with their usual status. Exercise was to be stopped, and medical staff contacted promptly if the thrill became markedly weaker or disappeared, swelling progressed, skin temperature decreased substantially or cyanosis appeared, severe pain or numbness worsened, incision bleeding or redness, warmth, and pain increased, or abnormal pulsation changed markedly. During the first 2 weeks after surgery, friction over the anastomotic area from equipment was avoided. If the incision showed bleeding or redness and swelling, fist clenching and ball squeezing were suspended and only gentle fingertip and opposition exercises were continued until reassessment. A rest period of 30 to 60 s was recommended between exercise modules to avoid local discomfort caused by continuous exertion.

Patients in the intervention group received one session of standardized exercise instruction during hospitalization. Research nurses used a unified script to explain the complete set of exercise movements, frequency, total duration per session, rules for staged progression, interruption principles, and recording requirements, and documented completion of instruction. Patients were also added on WeChat and informed that later exercise milestones would be prompted through WeChat reminders. Confirmation of key points on the day of discharge was conducted using a fixed set of questions to check understanding of exercise frequency and total duration per session, the way later exercise milestones would be triggered, and the principles for interruption and contacting the team, and the result was recorded. This step did not require patients to demonstrate the movements on site. At predetermined milestones, the research team sent WeChat reminders prompting patients to add the appropriate exercise module and conducted follow‐up at postoperative Weeks 2, 4, and 8 to check home exercise performance and discomfort, thereby aligning exercise progression with follow‐up time points.

### 2.7. Process Indicators

To quantify implementation feasibility and resource use, process indicators were recorded for the intervention group. Completion of standardized exercise instruction was defined as the presence of a complete instruction record, and the time required for standardized exercise instruction, in minutes, was recorded immediately by the research nurse after the one‐time inpatient teaching session. The result of confirmation of key points on the day of discharge was recorded as passed or not passed, and the type of key point requiring further explanation was categorized, such as exercise frequency and total duration per session, progression milestones, and interruption and contact principles. Home exercise adherence was collected by designated research nurses at postoperative Weeks 2, 4, and 8 using a low‐burden recall method. Patients were asked to report the number of completed sessions in the previous 7 days, ranging from 0 to 14, to describe adherence after discharge and its change over time. To reduce recall bias, patients were provided with a paper exercise record card and instructed to record each exercise session immediately after completion and to provide the card during follow‐up for reference.

### 2.8. Outcome Indicators and Data Collection

Data collected in this study included baseline characteristics, surgery‐related information, and outcome measures. The baseline questionnaire was developed on the basis of the literature review and previous work and covered age, sex, height, weight, body mass index, marital status, educational level, hypertension, diabetes, smoking, alcohol use, and exercise habits. Surgery‐related information was extracted from the hospital information system and included operative time, anastomosis type, duration of surgery, incision type, anastomotic characteristics, and vascular suture information.

The primary continuous outcome was BA flow at 8 weeks after surgery. The key clinical outcomes were time to maturation and maturation rate. Secondary ultrasound outcomes included draining vein (DV) diameter, RA diameter, and BA diameter. Safety outcomes included complication outcomes during follow‐up, such as stenosis, thrombosis, bleeding, and infection.

Ultrasound measurements were performed using color Doppler ultrasonography with a PHILIPS ultrasound system and an L12‐3Ns high‐frequency linear probe. Examinations were conducted by physicians who had received specialist training and had experience in vascular access assessment. Patients were placed in the supine position with the operated upper limb fully exposed. Ultrasound assessments were performed before surgery and 8 weeks after the intervention to quantify hemodynamic and vascular morphologic changes.

Maturation outcomes were determined by the hospital vascular access team on the basis of routine clinical assessment, with reference to the Chinese expert consensus on vascular access for hemodialysis [22]. AVF maturation was operationally defined as clinical usability for cannulation and was assessed through combined color Doppler ultrasound and physical examination. The assessment considered natural access flow ≥ 500 mL/min, venous diameter of the cannulation segment ≥ 5 mm, and cannulation‐segment depth from skin < 6 mm, together with physical examination findings, including a clearly palpable thrill over the anastomosis without abnormal enhancement, attenuation, or disappearance; a straight, superficial, uniform fistula vein with adequate cannulation area and good vessel wall elasticity; palpable thrill along the fistula segment; and no abnormal enhancement, attenuation, or disappearance of pulsation. The researchers themselves did not judge maturation; instead, they retrospectively extracted from the medical record the first date on which the AVF was documented as mature or usable for cannulation. Time to maturation was defined as the number of days from surgery to the first clinical record of a mature or usable AVF [23]. The 8‐week and 12‐week maturation rates were defined as maturation within 56 days and 84 days, respectively. Complication outcomes were followed for 12 weeks after the intervention. The timing of outcome assessment and data collection is shown in Table S5.

### 2.9. Blinding

Because the intervention involved structured exercise instruction, discharge confirmation, and follow‐up support, blinding of participants and intervention nurses was not feasible. However, ultrasound assessors and the vascular access team responsible for maturation assessment were blinded to group assignment.

### 2.10. Statistical Analysis

Quantitative data were analyzed using SPSS 27.0. Continuous variables were tested for normality using the Shapiro–Wilk test and for homogeneity of variance using Levene’s test. Variables that met the assumptions of normality and equal variance are presented as mean ± standard deviation and were compared using the independent‐samples *t* test. Variables that were not normally distributed are presented as median and interquartile range and were compared using the Mann–Whitney *U* test. Categorical variables are presented as frequency and percentage and were compared using the chi‐square test or Fisher’s exact test.

Within‐group comparisons of ultrasound outcomes before and after the intervention were performed using the Wilcoxon signed rank test. To reduce potential confounding, adjusted analyses for the main ultrasound outcomes and clinical maturation outcomes included age, sex, and diabetes as clinical covariates. For continuous ultrasound outcomes at 8 weeks, analysis of covariance was used, with the corresponding preoperative ultrasound value additionally included as a baseline covariate. Time to maturation was analyzed after natural log transformation using linear regression, and maturation rates were analyzed using binary logistic regression to estimate adjusted odds ratios. Hypertension was further included in sensitivity analyses to examine the robustness of the findings. All tests were two‐sided, and the significance level was set at *α* = 0.05. Delphi consultation data were summarized descriptively, and the expert authority coefficient and Kendall’s coefficient of concordance were calculated and tested to assess the degree of agreement.

### 2.11. Ethics

The study was conducted in accordance with the principles of the Declaration of Helsinki. The study protocol was approved by the Medical Ethics Committee of the Second Affiliated Hospital of Guangzhou University of Chinese Medicine (Approval No. ZF2024‐030‐01).

## 3. Results

### 3.1. Evidence Review Results

Focusing on upper limb and hand exercises to promote AVF maturation after surgery in patients requiring hemodialysis, eight randomized controlled trials involving 571 patients were ultimately included. Three broad forms of intervention were identified. The first consisted of prescribed isometric exercise and handgrip or ball‐squeezing exercise, usually with dose defined by a fixed number of repetitions, sets, and duration, and with fistula maturation objectively assessed using Doppler ultrasound parameters such as blood flow and vessel diameter together with maturation time and maturation rate. Some studies used staged progression strategies, whereas others compared different devices or exercise intensities to explore the relationship between exercise dose, hemodynamic improvement, and maturation outcomes [9, 10, 24, 25]. The second type combined exercise training with upper arm tourniquet or band compression to investigate additional benefits for hemodynamic and maturation‐related indicators [10, 12]. The third type used a rehabilitation exercise format in which movements were organized into a fixed routine and audio‐visual instruction or follow‐up support was incorporated to strengthen patient understanding and consistency of execution [26–28].

### 3.2. Results of the Delphi Expert Consultation

After Round 1, the research group revised the indicator framework in light of expert feedback and developed the Round 2 questionnaire. Three third‐level indicators (1.2.3, 2.1.3, and 2.3.3) were deleted, and two third‐level indicators were merged, with 2.2.4 incorporated into 2.2.3 and 2.5.3 incorporated into 2.5.2. Item allocation was also adjusted, with 3.3.1 moved to 4.2.3 and rewritten accordingly. In addition, the first‐level structure was modified by replacing the original first‐level indicator “V. Nurses’ management of patients” with “V. Empowerment and long‐term management.” Under this new first‐level indicator, three second‐level indicators (5.1, 5.2, and 5.3) and four third‐level indicators (5.2.3, 5.3.1, 5.3.2, and 5.3.3) were added. Item wording was also standardized, with expressions such as “nurse teaches” revised into patient‐centered wording such as “the patient is able to,” “understands,” or “masters.” The final indicator system after Round 2 comprised 5 first‐level indicators, 16 second‐level indicators, and 38 third‐level indicators.

Across the two rounds, expert authority was high, with expert authority coefficients (Cr) exceeding 0.90 in both rounds. Importance ratings at all levels were generally high, and the degree of dispersion was low. In Round 1, mean importance scores ranged from 4.20 to 4.60 for first‐level indicators, with coefficients of variation from 0.11 to 0.13; from 4.13 to 4.87 for second‐level indicators, with coefficients of variation from 0.07 to 0.18; and from 3.67 to 4.93 for third‐level indicators, with coefficients of variation from 0.05 to 0.25 (Table S6). In Round 2, the corresponding ranges were 4.21 to 5.00 with coefficients of variation from 0.00 to 0.10 for first‐level indicators, 4.07 to 4.86 with coefficients of variation from 0.07 to 0.15 for second‐level indicators, and 4.07 to 4.93 with coefficients of variation from 0.05 to 0.15 for third‐level indicators (Table 2). In Round 2, Kendall’s coefficient of concordance was 0.271 (*p* < 0.001).

**TABLE 2:** Results of Round 2 Delphi expert consultation.

Item	Importance score, mean ± SD	CV
I. Patient eligibility and initial assessment	5.00 ± 0.00	0.00
1.1 Surgical and fistula assessment	4.86 ± 0.36	0.07
1.1.1 Confirm that the patient meets the starting time for exercise at this stage	4.79 ± 0.58	0.12
1.1.2 No active bleeding or obvious hematoma at the fistula anastomosis	4.93 ± 0.27	0.05
1.1.3 Slight thrill can be palpated or a faint bruit can be heard over the fistula vessel	4.79 ± 0.43	0.09
1.2 Assessment of baseline patient status	4.57 ± 0.51	0.11
1.2.1 The patient is conscious and able to understand and follow instructions	4.64 ± 0.50	0.11
1.2.2 Normal skin temperature, color, and sensation of the operated hand, with no severe edema	4.57 ± 0.51	0.11
1.3 Assessment of patient willingness and support system	4.29 ± 0.61	0.14
1.3.1 Patient willingness to participate in exercise	4.43 ± 0.65	0.15
1.3.2 Feasibility of family supervision and assistance (including receiving/relaying WeChat milestone reminders, checking exercise progression key points, and assisting with recording)	4.29 ± 0.47	0.11
II. Implementation of the staged exercise program	4.21 ± 0.43	0.10
2.1 Postoperative 12–24 h (positioning phase)	4.79 ± 0.43	0.09
2.1.1 The patient can maintain the operated upper limb in the correct elevated position	4.43 ± 0.51	0.12
2.1.2 The patient can maintain slight elbow flexion and a neutral wrist position to avoid pressure on the anastomosis	4.29 ± 0.47	0.11
2.2 Postoperative Day 2 (fingertip activity phase)	4.07 ± 0.62	0.15
2.2.1 The patient can correctly demonstrate piano‐like fingertip tapping	4.93 ± 0.27	0.05
2.2.2 The patient can correctly demonstrate opposition exercise	4.29 ± 0.47	0.11
2.2.3 The patient understands and agrees to follow an exercise frequency of once each morning and evening with an interval of ≥ 8 h and can complete the session at fixed times with WeChat reminders or self‐set alarms	4.79 ± 0.43	0.09
2.3 Postoperative Days 3–6 (wrist and fist‐clenching phase)	4.71 ± 0.47	0.10
2.3.1 The patient can correctly and completely demonstrate the full wrist movement sequence	4.93 ± 0.27	0.05
2.3.2 The patient can correctly perform “empty fist‐full hand opening” and “suspended wrist fist clenching”	4.71 ± 0.47	0.10
2.4 Postoperative Days 7–14 (ball‐squeezing strengthening phase)	4.43 ± 0.65	0.15
2.4.1 The patient can correctly complete the cycle “squeeze for 5 s ⟶ relax for 5 s”	4.50 ± 0.65	0.14
2.4.2 The patient can perform the elbow flexion ball‐squeezing movement in a coordinated manner	4.43 ± 0.65	0.15
2.4.3 The patient knows how to adjust exercise intensity according to soreness or tightness	4.07 ± 0.27	0.07
2.5 Postoperative Day 14 to Week 8 (consolidation and progression phase)	4.29 ± 0.61	0.14
2.5.1 The patient can independently complete the full “warm‐up, core, relaxation” sequence	4.43 ± 0.51	0.12
2.5.2 Under nurse guidance and based on follow‐up checks and WeChat feedback, the patient can safely progress exercise intensity according to the program	4.29 ± 0.47	0.11
III. Monitoring and adjustment during exercise	4.93 ± 0.27	0.05
3.1 Monitoring of intensity and tolerance	4.14 ± 0.36	0.09
3.1.1 The nurse confirms that exercise intensity always complies with the principle of “able to speak comfortably without breath‐holding”	4.29 ± 0.47	0.11
3.1.2 Observe and record the patient’s subjective responses after exercise (e.g., soreness, numbness, tightness, and pain)	4.86 ± 0.36	0.07
3.2 Monitoring of fistula status and complications	4.79 ± 0.43	0.09
3.2.1 Assess whether fistula thrill and bruit remain satisfactory before each exercise session	4.79 ± 0.43	0.09
3.2.2 Monitor and record complications such as arm swelling, subcutaneous hematoma, and anastomotic bleeding	4.86 ± 0.36	0.07
3.2.3 Emergency response plan for abnormalities during exercise (e.g., weakened/disappeared thrill or bruit, severe pain)	4.93 ± 0.27	0.05
3.3 Adherence monitoring	4.21 ± 0.43	0.10
3.3.1 Use the Record Card together with WeChat reminders/feedback to calculate the ratio of actual weekly exercise sessions to the target number (14), and record reasons for missed sessions when necessary to support follow‐up adjustment	4.71 ± 0.47	0.10
IV. Health education and management	4.79 ± 0.43	0.09
4.1 Knowledge education	4.29 ± 0.47	0.11
4.1.1 The patient can restate the main purpose of exercise at each stage	4.21 ± 0.43	0.10
4.1.2 The patient understands the overall duration and stage‐based structure of the exercise program	4.21 ± 0.58	0.14
4.2 Behavioral guidance	4.14 ± 0.36	0.09
4.2.1 Before discharge, the patient confirms feasible fixed exercise times/settings (e.g., morning and evening) with the nurse and agrees to follow the prescribed exercise frequency and duration (about 3 min per movement and no more than 25 min per session)	4.36 ± 0.63	0.15
4.2.2 The patient can identify at least two “red‐flag” signs that require immediate cessation of exercise and reporting	4.21 ± 0.43	0.10
4.2.3 The patient/family can independently complete the hand exercise record card	4.71 ± 0.47	0.10
V. Empowerment and long‐term management	4.21 ± 0.43	0.10
5.1 Mastery of knowledge and skills	4.64 ± 0.50	0.11
5.1.1 Before discharge, the patient can independently restate the key points and precautions of the full program	4.36 ± 0.50	0.11
5.1.2 The patient can correctly demonstrate all core movements for the current stage and previous stages	4.29 ± 0.47	0.11
5.2 Self‐management and problem‐solving	4.43 ± 0.51	0.12
5.2.1 The patient can self‐monitor using the record card and has a plan to make up for missed exercise	4.43 ± 0.51	0.12
5.2.2 The patient knows how to respond to common problems such as mild soreness or forgetting the movements	4.36 ± 0.63	0.15
5.2.3 The patient clearly knows under which circumstances medical staff should be contacted proactively	4.93 ± 0.27	0.05
5.3 Long‐term behavioral maintenance and confidence building	4.43 ± 0.65	0.15
5.3.1 After discharge, the patient can maintain high exercise adherence (> 80%) through follow‐up feedback	4.64 ± 0.50	0.11
5.3.2 With clear safety boundaries and a pathway for seeking care, the patient feels confident about maintaining exercise for 8 weeks and promoting AVF maturation	4.71 ± 0.47	0.10
5.3.3 The patient can regard this exercise as a long‐term self‐management behavior that is as important as dialysis treatment	4.79 ± 0.43	0.09

*Note:* CV, coefficient of variation.

Abbreviation: SD, standard deviation.

### 3.3. Study Implementation and Process Indicators

A total of 88 patients were invited across the two study phases. In the usual care phase, all 41 invited patients were enrolled, received usual care, completed the 8‐week follow‐up, and were included in the analysis. In the intervention phase, 47 patients were invited; 6 withdrew before intervention initiation for personal or logistical reasons, and the remaining 41 were enrolled, received the intervention, completed the 8‐week follow‐up, and were included in the analysis. No included participant was lost to follow‐up.

All 41 patients in the intervention group completed standardized exercise instruction during hospitalization and had a corresponding record, giving a completion rate of 100.0%. The mean time required for standardized exercise instruction was 12.4 ± 3.1 min, describing the bedside teaching input required in routine ward practice. On the day of discharge, 34 patients (82.9%) passed confirmation of key points at the first attempt, whereas 7 (17.1%) passed after additional explanation. The main issues requiring further explanation were exercise frequency and total duration per session in 5 cases, the method used to trigger exercise progression milestones through WeChat reminders in 4 cases, and principles for interruption and contacting the team in 3 cases; the same patient could require further explanation on more than one item. Home exercise adherence, recorded as the number of completed sessions in the past 7 days, was 10 (8, 12), 11 (9, 13), and 12 (10, 14) at postoperative Weeks 2, 4, and 8, respectively (Table 3). These process indicators provide preliminary implementation information on ward teaching workload, discharge transition support, and postdischarge adherence continuity, which may inform the assessment of the organizational sustainability of the nurse‐led program.

**TABLE 3:** Implementation feasibility and process indicators of the staged exercise management program (intervention group).

Dimension	Indicator	Result
Standardized exercise instruction	Completion of standardized exercise instruction, *n* (%)	41 (100.0%)
	Time required for standardized exercise instruction, min	12.4 ± 3.1

Discharge‐day confirmation of key points	Passed at first verification, *n* (%)	34 (82.9%)
	Passed after additional explanation, *n* (%)	7 (17.1%)
	Main key points requiring additional explanation^∗^	Exercise frequency and total duration per session (5 cases); triggering method for exercise progression milestones (WeChat reminders) (4 cases); interruption and contact principles (3 cases)

Home exercise adherence (number of completed sessions in the past 7 days)	Postoperative Week 2, median (Q1, Q3)	10 (8.12)
	Postoperative Week 4, median (Q1, Q3)	11 (9.13)
	Postoperative Week 8, median (Q1, Q3)	12 (10.14)

^∗^More than one item could require additional explanation for the same patient.

### 3.4. Baseline Characteristics

All participants were right‐handed and therefore underwent AVF creation in the left forearm. The mean age of the intervention group was 51.02 ± 10.85 years, and 28 participants (68.3%) were male; the mean age of the control group was 50.73 ± 10.23 years, and 24 participants (58.5%) were male. No statistically significant differences were observed between groups in age, height, weight, body mass index, sex, marital status, educational level, hypertension, diabetes, smoking, alcohol use, or exercise habits (*p* > 0.05; Table 4). Given the nonrandomized, phase‐based allocation design, analyses of both primary and secondary outcomes were adjusted for prespecified covariates.

**TABLE 4:** Comparison of baseline characteristics between the two groups.

Variable	Control group	Intervention group	*t*/*χ* ^2^	*p*
	(*n* = 41)	(*n* = 41)		
Age	50.73 ± 10.23	51.02 ± 10.85	−0.126	0.900^a^
Height (cm)	161.05 ± 7.68	163.93 ± 8.40	−1.620	0.109^a^
Weight (kg)	64.26 ± 13.62	67.81 ± 15.29	−1.111	0.270^a^
BMI (kg/m^2^)	24.68 ± 4.55	25.07 ± 4.75	−0.377	0.707^a^
Sex (%)			0.841	0.359^b^
Male	24 (58.5%)	28 (68.3%)		
Female	17 (41.5%)	13 (31.7%)		
Marital status (%)			—	0.057^c^
Married	40 (97.6%)	34 (82.9%)		
Unmarried and others	1 (2.4%)	7 (17.1%)		
Educational level (%)			4.089	0.252^b^
Primary school and below	11 (26.8%)	13 (31.7%)		
Junior high school	15 (36.6%)	7 (17.1%)		
Senior high school and secondary technical school	9 (22.0%)	13 (31.7%)		
College and above	6 (14.6%)	8 (19.5%)		
Hypertension (%)			—	0.359^c^
Yes	40 (97.6%)	37 (90.2%)		
No	1 (2.4%)	4 (9.8%)		
Diabetes (%)			3.216	0.073^b^
Yes	28 (68.3%)	20 (48.8%)		
No	13 (31.7%)	21 (51.2%)		
Smoking (%)			1.439	0.230^b^
Yes	10 (24.4%)	15 (36.6%)		
No	31 (75.6%)	26 (63.4%)		
Alcohol (%)			—	0.264^c^
Yes	6 (14.6%)	2 (4.9%)		
No	35 (85.4%)	39 (95.1%)		
Exercise habit (%)			0.345	0.557^b^
Yes	6 (14.6%)	8 (19.5%)		
No	35 (85.4%)	33 (80.5%)		

Abbreviation: BMI, body mass index.

^a^independent‐samples *t* test.

^b^chi‐square test.

^c^Fisher’s exact test.

### 3.5. Preoperative Ultrasound and Surgery‐Related Indicators

All operations were performed by the same surgeon, using an oblique incision of approximately 5 cm and an end‐to‐side anastomosis with 8‐0 atraumatic vascular sutures. Preoperative ultrasound indicators and surgery‐related indicators were broadly comparable between the two groups (*p* > 0.05; Table 5).

**TABLE 5:** Comparison of preoperative ultrasound and surgery‐related indicators between the two groups.

Variable	Control group	Intervention group	*χ* ^2^/*Z*	*p*
BA flow (mL/min)	337.67 (244.28, 443.59)	304.98 (259.79, 380.10)	−1.164	0.244^b^
BA diameter (mm)	3.90 (3.50, 4.30)	4.20 (4.00, 4.85)	−0.037	0.970^b^
RA diameter (mm)	2.10 (1.90, 2.55)	2.20 (1.90, 2.45)	−0.228	0.820^b^
RA PSV (cm/s)	64.00 (55.50, 71.00)	59.00 (49.50, 74.00)	−0.668	0.504^b^
UA diameter (mm)	1.80 (1.55, 2.10)	2.00 (1.70, 2.50)	−1.941	0.052^b^
UA PSV (cm/s)	63.80 (47.00, 74.00)	56.00 (46.00, 67.00)	−1.011	0.312^b^
DV diameter (mm)	2.20 (1.75, 2.60)	2.50 (1.75, 2.95)	−1.616	0.106^b^
Operation time (min)	81.00 (71.50, 90.00)	75.00 (60.0, 89.50)	−1.518	0.129^b^
Anastomosis length (cm)			0.648	0.723^a^
0.6	12 (29.3%)	9 (22.0%)		
0.7	10 (24.4%)	10 (24.4%)		
0.8	19 (46.3%)	22 (53.7%)		

Abbreviations: BA, brachial artery; DV, draining vein; PSV, peak systolic velocity; RA, radial artery; UA, ulnar artery.

^a^chi‐square test.

^b^Mann–Whitney *U* test.

### 3.6. Between‐Group Comparison of Ultrasound Outcomes

At 8 weeks after the intervention, ultrasound outcomes were generally more favorable in the intervention group than in the control group, with the most marked between‐group difference seen in the primary continuous ultrasound outcome, BA flow. In the main adjusted model, BA flow in the intervention group was 137.210 mL/min higher than in the control group (95% CI 62.857–211.564, *p* < 0.001), suggesting that the staged exercise management program was associated with a more favorable hemodynamic profile of the vascular access. In addition, BA diameter and RA diameter were larger in the intervention group, with adjusted between‐group differences of 0.405 mm (95% CI 0.112–0.698, *p* = 0.007) and 0.256 mm (95% CI 0.013–0.499, *p* = 0.039), respectively. By contrast, although DV diameter tended to be larger in the intervention group, the adjusted difference did not reach statistical significance (*β* = 0.376 mm, 95% CI −0.058–0.810, *p* = 0.088). After additional adjustment for hypertension in sensitivity analyses, the direction and magnitude of the between‐group differences for BA flow, BA diameter, and RA diameter remained broadly unchanged, supporting the robustness of the findings (Table 6).

**TABLE 6:** Between‐group comparison of ultrasound outcomes at 8 weeks after implementation of the staged exercise management program.

Outcome	Model	Control group	Intervention group	*β*	95% CI	*p*
BA flow	Crude	746.10 (615.64, 883.15)	838.49 (718.66, 960.01)	109.555	31.903–187.207	0.006
	Model 1	—	—	134.591	63.312–205.870	< 0.001
	Model 2	—	—	133.189	60.544–205.835	< 0.001
	**Model 3**	**—**	**—**	**137.210**	**62.857–211.564**	**<** **0.001**
	Sensitivity model	—	—	145.674	70.649–220.698	< 0.001

BA diameter	Crude	5.30 (4.85, 5.60)	5.80 (5.00, 6.25)	0.510	0.190–0.829	0.002
	Model 1	—	—	0.510	0.212–0.807	0.001
	Model 2	—	—	0.480	0.185–0.775	0.002
	**Model 3**	**—**	**—**	**0.405**	**0.112–0.698**	**0.007**
	Sensitivity model	—	—	0.461	0.172–0.750	0.002

RA diameter	Crude	4.00 (3.60, 4.55)	4.40 (4.10, 4.75)	0.368	0.113–0.624	0.005
	Model 1	—	—	0.354	0.101–0.607	0.007
	Model 2	—	—	0.336	0.084–0.588	0.010
	**Model 3**	**—**	**—**	**0.256**	**0.013–0.499**	**0.039**
	Sensitivity model	—	—	0.283	0.038–0.528	0.024

DV diameter	Crude	5.00 (4.15, 5.85)	5.20 (4.70, 6.00)	0.424	0.013–0.835	0.043
	Model 1	—	—	0.421	0.002–0.840	0.049
	Model 2	—	—	0.425	−0.001–0.851	0.050
	**Model 3**	**—**	**—**	**0.376**	**−0.058–0.810**	**0.088**
	Sensitivity model	—	—	0.377	−0.066–0.820	0.094

*Note:* Crude, unadjusted. Model 1, adjusted for the corresponding preoperative baseline ultrasound measure (BA blood flow was adjusted for preoperative BA blood flow; BA diameter was adjusted for preoperative BA diameter; RA diameter was adjusted for preoperative RA diameter; and DV diameter was adjusted for preoperative DV diameter). Model 2, based on Model 1 with additional adjustment for age and sex. Model 3, based on Model 2 with additional adjustment for diabetes. Sensitivity model, based on Model 3 with additional adjustment for hypertension. β represents the unstandardized regression coefficient. The bold values indicate the results of the prespecified main adjusted model (Model 3), which was selected as the primary model for interpretation.

Abbreviations: BA, brachial artery; CI, confidence interval; DV, draining vein; RA, radial artery.

### 3.7. Ultrasound Outcomes: Within‐Group Before and After Changes and Between‐Group Comparisons of Change Scores

From baseline to 8 weeks after the intervention, BA flow and BA, RA, and DV diameters all increased significantly from baseline in both groups (all within‐group *p* < 0.001). In the between‐group comparison of change scores, defined as the value at postoperative Week 8 minus the preoperative baseline value, the intervention group showed larger increases in BA flow and BA diameter than the control group, suggesting more favorable observed changes in blood flow and vascular diameter. By contrast, the between‐group difference in the change in DV diameter was not statistically significant, whereas the difference in the change in RA diameter was only borderline significant (Table S7).

### 3.8. AVF Maturation Outcomes

The intervention group outperformed the control group in both AVF maturation rate and speed of maturation. At postoperative Week 8, the maturation rate was 95.1% (39/41) in the intervention group and 75.6% (31/41) in the control group (*p* = 0.012). After adjustment for age, sex, and diabetes, the likelihood of achieving maturation by Week 8 remained significantly higher in the intervention group than in the control group (aOR = 7.706, 95% CI 1.453–40.873, *p* = 0.016); the result was essentially unchanged after further adjustment for hypertension. At postoperative Week 12, the maturation rate was 100.0% (41/41) in the intervention group and 82.9% (34/41) in the control group, and the between‐group difference remained statistically significant (Fisher’s exact test, *p* = 0.012). Because no patient in the intervention group remained nonmature at Week 12, adjusted odds ratios were not reported. Time to maturation was shorter in the intervention group than in the control group at 49.00 (44.00, 54.50) days versus 55.00 (52.00, 58.00) days (*p* < 0.001). After adjustment, time to maturation remained significantly shorter in the intervention group (TR = 0.81, 95% CI 0.73–0.90, *p* < 0.001), and the result was unchanged in sensitivity analysis (Table 7).

**TABLE 7:** Comparison of AVF maturation‐related clinical outcomes.

Outcome	Model	Control group	Intervention group	Effect	95% CI	*p*
Week‐8 maturation, *n*/*N* (%)	Unadjusted	31/41 (75.6%)	39/41 (95.1%)	*χ* ^2^ = 6.248	—	0.012
	Adjusted	—	—	aOR = 7.706	1.453–40.873	0.016
	Sensitivity model	—	—	aOR = 7.501	1.381–40.743	0.020

Week‐12 maturation, *n*/*N* (%)	Unadjusted	34/41 (82.9%)	41/41 (100.0%)	—	—	0.012^a^
	Adjusted	—	—	—^b^	—	—

Time to maturation, days	Unadjusted	55.00 (52.00, 58.00)	49.00 (44.00, 54.50)	*Z* = −3.523	—	< 0.001
	Adjusted	—	—	TR = 0.81	0.73–0.90	< 0.001
	Sensitivity model	—	—	TR = 0.82	0.73–0.91	< 0.001

*Note:* For Week‐8 maturation and time to maturation, the adjusted models included age, sex, and diabetes, and the sensitivity models additionally included hypertension. For Week‐12 maturation, only the unadjusted comparison was reported; the adjusted odds ratio was not estimable because all AVFs in the intervention group had matured by Week 12, resulting in complete separation in standard logistic regression. TR, time ratio, calculated as exp (*B*) from the linear regression model of ln‐transformed time to maturation, with the control group as the reference; a TR < 1 indicates that the intervention group had a shorter time to maturation than the control group. AVF, arteriovenous fistula.

Abbreviations: aOR, adjusted odds ratio; CI, confidence interval.

^a^Fisher’s exact test.

^b^Not estimable.

### 3.9. Safety: Complication Outcomes

During the 8‐week follow‐up period, only stenosis and thrombosis were observed; no bleeding or infection events occurred. The overall complication rate did not differ significantly between groups (*p* > 0.05). Continued follow‐up to 12 weeks showed that all AVFs in the intervention group had matured and that no additional complications were observed (Table 8).

**TABLE 8:** Complication rates through 8 weeks of follow‐up, *n* (%).

Variable	*n*	Stenosis	Thrombosis	Overall complication rate	*p*
Intervention group	41	3 (7.3)	1 (2.4)	4 (9.7)	0.564^a^
Control group	41	6 (14.6)	1 (2.4)	7 (17.1)	

^a^Fisher’s exact test.

## 4. Discussion

This study addressed the clinically important issue of early functional establishment of autogenous AVFs in patients requiring hemodialysis. Based on a structured evidence review, Delphi expert consultation, and pilot testing, a nurse‐led perioperative staged exercise management program was developed and its short‐term effects were evaluated in a single‐center prospective controlled study. The Delphi consultation showed high expert authority and acceptable consensus, supporting the content validity and clinical acceptability of the program. In the controlled evaluation, the intervention group showed more favorable early ultrasound outcomes and maturation‐related clinical outcomes than the usual care group. The ultrasound findings suggest that the staged exercise management program may be associated with a more favorable hemodynamic profile and selected vascular enlargement, particularly in relation to BA blood flow and arterial diameter. By contrast, the adjusted between‐group difference in DV diameter did not reach statistical significance, suggesting that vascular morphologic responses to exercise management may not be uniform across indicators. Clinically, earlier achievement of AVF maturation was observed in the intervention group, with no observed increase in complications during follow‐up. Process indicators further suggested feasibility in ward practice, including high completion of standardized instruction, manageable instruction time, and maintained home exercise adherence. Overall, the nurse‐led staged exercise management program developed and implemented in this study was associated with faster achievement of clinical usability and a higher rate of early maturation, and it offers a reproducible implementation pathway for perioperative AVF nursing management [11, 29].

From a physiologic perspective, regular and progressive contraction of the hand and forearm muscles may influence the local hemodynamic environment and vascular remodeling through several pathways and thereby contribute to faster AVF maturation [30–33]. On the one hand, exercise‐induced muscle pump effects and enhanced flow‐related shear stress may activate endothelial function and promote vascular dilation and remodeling, creating a hemodynamic environment that is more favorable for early maturation [11, 28, 30]. On the other hand, after creation of an arteriovenous shunt, the proximal arterial system may undergo adaptive enlargement and increased blood flow under sustained high‐flow stimulation, which may help establish a more stable hemodynamic environment within the access [25, 34]. This mechanistic interpretation is broadly consistent with the present findings: The more favorable maturation‐related outcomes observed in the intervention group, together with the direction of change in BA flow, BA diameter, and RA diameter, appeared consistent with a more favorable access hemodynamic profile and selected vascular enlargement, suggesting that the main advantage of the staged exercise program may be its ability to help the AVF become clinically usable more quickly. The staged strategy of early safety, midterm functional recovery, and later consolidation and progression allowed exercise load to match the postoperative recovery trajectory. In the early phase, postural management and gentle fingertip activity were emphasized to reduce the risk of traction at the anastomosis and excessive early loading. During recovery, wrist movements, fist clenching, and ball squeezing, which have clearer resistance characteristics, were introduced gradually to provide sustained and cumulative hemodynamic stimulation, in keeping with the process of adaptive outward remodeling in AVFs under high‐flow conditions [9, 31, 33]. The combination of staged progression, standardized exercise instruction, and follow‐up checks may also have improved the reproducibility and traceability of the intervention in clinical practice.

The COM‐B model, which includes capability, opportunity, motivation, and behavior, provided the theoretical framework for organizing the intervention components and implementation strategies in this study [16]. At the capability level, standardized exercise instruction during hospitalization, confirmation of key points on the day of discharge, and targeted supplementary explanation may have improved patients’ understanding of and ability to carry out the required exercise frequency, total duration per session, progression milestones, and interruption principles [35]. At the opportunity level, WeChat milestone reminders and checks at fixed follow‐up time points made exercise progression and review more accessible as process support, reducing forgetfulness, delay, and interruption and making home exercise easier to incorporate into everyday life [36, 37]. At the motivation level, the staged progression schedule and explicit safety boundaries may have eased concerns about exercise after surgery, performing movements incorrectly, or harming the fistula, thereby strengthening willingness to continue [38]. Consistent with this framework, the study also produced process evidence regarding implementation feasibility and behavior attainment. Completion of standardized exercise instruction in the intervention group was 100.0%, and a single session required only 12.4 ± 3.1 min, suggesting that stable, low‐burden instruction was feasible in a routine ward workflow. In confirmation of key points on the day of discharge, 82.9% of patients passed on the first attempt and 17.1% passed after additional explanation. The issues most often requiring clarification involved exercise frequency and total duration per session, the way exercise progression milestones were triggered through WeChat reminders, and the principles for interruption and contact, indicating that these key points can be standardized and corrected through structured verification and supplementary explanation [33]. Home exercise adherence, assessed using a low‐burden recall method, increased over time, with the number of completed sessions in the past 7 days rising from 10 at Week 2 to 11 at Week 4 and 12 at Week 8. Taken together, the high completion of standardized instruction, completion of confirmation of key points, and gradually increasing adherence suggest that the improvements observed in ultrasound and maturation outcomes were achieved not merely because a program existed, but on the basis of relatively consistent instruction and a degree of sustained performance at home. These process indicators support the implementation feasibility of the program and the enactment of exercise behavior; however, because home exercise adherence was measured using a low‐burden recall method and no objective monitoring of movement quality or actual exercise dose was conducted, execution bias cannot be ruled out [18, 39].

From a nursing management perspective, the value of this program lies primarily in advancing postoperative AVF exercise guidance from the simple addition of exercise content to a nurse‐led management pathway that can be implemented through standardized exercise instruction, while integrating exercise prescription, confirmation of key points, milestone reminders, and follow‐up review into a continuous process. Compared with one‐time, generic discharge education, this pathway places greater emphasis on specifying exercise requirements, standardizing exercise instruction, and identifying patients’ misunderstandings or concerns regarding exercise safety, progression timing, and management of abnormal situations during continuous support. In this way, nurses are better able to support patients’ understanding of the training content, enactment of exercise behavior, and long‐term self‐management [13, 40]. Based on these assessment findings and identified problems, nurses can translate the staged exercise requirements into a concrete guidance plan and promote implementation through discharge‐day confirmation, stage‐based reminders, and follow‐up review. Subsequent adherence review, ultrasound assessment, maturation assessment, and complication monitoring provide feedback for continuously judging patients’ behavioral performance and AVF functional establishment. This process is consistent with the role of the nursing process in clinical decision‐making, nursing documentation, and outcome evaluation and may help transform nursing activities from one‐time education into an executable, traceable, and evaluable management process [41]. Through this management loop, nurses’ responsibilities in health education, risk identification, behavioral support, follow‐up coordination, and outcome monitoring become clearer, which may help strengthen their autonomy and leadership in perioperative vascular access care.

This is broadly consistent with evidence, suggesting that structured nurse‐led interventions can improve adherence, self‐management, and selected health outcomes across chronic care settings [42]. Recent studies suggest that the quality of communication between patients and providers influences self‐management among people with chronic illness and that barriers and facilitators related to self‐care and disease management in dialysis patients are highly individualized. This indicates that nursing strategies should be designed as ongoing processes built around understanding, support, and participation [14, 43]. In addition, the latest vascular access guidelines emphasize that standards for vascular access care apply not only to physicians but also to hemodialysis nurses, and that vascular access management should extend beyond surgical creation to include perioperative care, monitoring, and ongoing management [4]. Behavioral and human factors also shape this process, and behavioral frameworks such as COM‐B may help organize relevant findings and support the optimization of clinical workflows [44]. Given the high completion of standardized instruction, the high rate of successful confirmation of key points, and the favorable home exercise adherence observed in this study, the program appears not only operationally feasible but also reasonably consistent in implementation. By operationalizing the nursing process within a continuous perioperative management pathway, the program may contribute to more standardized AVF nursing care and provide a practical basis for future institutional protocol development. Its main significance lies in providing a reproducible, traceable, and continuity‐oriented approach to nurse‐led perioperative AVF management.

Compared with previous studies, this study further linked exercise prescription with an inpatient‐to‐home management pathway and formed a process‐based nursing intervention that can be prompted, checked, and recorded. Previous studies on hand exercise suggest that hand or upper limb exercise may improve ultrasound indicators related to AVF maturation, including blood flow, vessel diameter, and cephalic vein caliber, which is consistent with the more favorable direction of BA blood flow and arterial diameter observed in the intervention group in the present study [29, 45]. At the same time, the Hemodialysis Fistula Maturation Study, a seven‐center study conducted in the United States, showed that postoperative ultrasound measures, including blood flow, diameter, and depth, had only moderate predictive ability for clinical maturation [46]; therefore, the nonsignificant adjusted between‐group difference in DV diameter in this study suggests that a single ultrasound indicator may not fully reflect the clinical maturation process. Previous studies of ball squeezing, isometric training, or comprehensive rehabilitation exercise have focused mainly on exercise formats and ultrasound changes, whereas less attention has been paid to implementation issues such as how to deliver standardized exercise instruction during hospitalization, how to define clear time points for exercise progression, how to maintain and review performance after discharge using low‐burden methods, and how to record process data to help explain outcomes [11, 23, 29]. In this study, what was added to usual care was a composite management program that combined a staged exercise program with structured implementation support. Its distinctiveness lay both in the systematic design of movements, timing of initiation, staged progression, and exercise dose, and in practical supports such as standardized exercise instruction during hospitalization, confirmation of key points on the day of discharge, WeChat milestone reminders, and follow‐up checks, all of which are feasible in ordinary wards. Future studies could further examine its external applicability in multiple centers and across different populations, surgical techniques, and follow‐up conditions, and optimize reminder frequency, progression milestones, and individualized dosing strategies without increasing burden. They could also explore potential effects on longer term outcomes such as successful cannulation, long‐term patency, and reintervention rates, thereby generating more systematic evidence for perioperative AVF function maintenance.

## 5. Limitations

This study has several limitations that warrant cautious interpretation. First, it was a single‐center prospective controlled study in which participants were enrolled and allocated by study phase. Although this design helped reduce contamination caused by health education within the ward, it may have introduced temporal bias, such as changes in ward workflow or increasing proficiency of the education team over time. The causal strength of the evidence is therefore lower than that of a strictly individual randomized design. Future studies should further validate the effectiveness and generalizability of this program through multicenter randomized controlled trials, cluster randomized designs, stepped‐wedge designs, or stronger quasi‐experimental designs.

Second, follow‐up mainly covered 8 to 12 weeks after surgery and time to maturation for all patients. Although this time frame can reflect early hemodynamic improvement and maturation outcomes, it is insufficient to evaluate longer term and more clinically meaningful end points such as long‐term patency, successful cannulation, and reintervention rates. In addition, management‐related outcomes, including hospital costs, length of stay, readmission, care transitions, and resource use, were not collected. This limited our ability to evaluate the broader organizational and economic impact of the nurse‐led pathway. Future studies should extend the follow‐up period and include long‐term vascular access outcomes, management‐related outcomes, and cost‐related indicators.

Third, home exercise adherence was obtained using a low‐burden recall method. Although this approach was feasible for real‐world follow‐up, it may have been subject to recall bias or social desirability bias. In addition, movement quality and the consistency with which the intended exercise dose was achieved were not objectively quantified, for example, through video checks, wearable monitoring, or device‐based resistance or handgrip dose recording. This limited a fine‐grained analysis of the dose–response relationship among actual exercise dose, ultrasound changes, and maturation outcomes. Future studies could combine objective monitoring tools with process evaluation methods to improve the accuracy of home exercise performance assessment.

Finally, COM‐B was used as a theoretical reference for intervention development and implementation design, but the study did not include comparison conditions that would allow the independent contribution of each behavioral support component, including standardized instruction, confirmation of key points, WeChat reminders, and follow‐up checks, to be separated. Accordingly, the relative contribution of each component to the observed improvements cannot be determined. Future studies may use component analysis, factorial designs, or process evaluation to further examine the mechanisms of different intervention components.

## 6. Contributions to the Advancement of Knowledge

This study contributes to the development of perioperative AVF nursing knowledge by integrating fragmented postoperative exercise guidance into a structured nurse‐led management pathway. It adds to existing exercise‐focused literature by showing that AVF maturation support can be organized as a continuous pathway linking patient education, behavioral maintenance, safety monitoring, and outcome feedback. The study also provides preliminary implementation knowledge by reporting process indicators alongside ultrasound and maturation outcomes, thereby helping to explain how the intervention was delivered and maintained in routine ward practice. In addition, it demonstrates how the nursing process can be operationalized in perioperative vascular access care, offering a conceptual basis for future standardized ward procedures, institutional protocols, and multicenter studies of sustained postoperative nursing management.

## 7. Conclusion

The results of this study indicate that, when added to usual care, a nurse‐led perioperative staged exercise management program that combines standardized exercise instruction during hospitalization, confirmation of key points on the day of discharge, WeChat milestone reminders, and follow‐up checks was associated with more favorable early ultrasound and maturation‐related outcomes after AVF creation. Taken together, these findings suggest that embedding staged exercise guidance within a structured nurse‐led management pathway may support early AVF functional establishment while maintaining feasibility in routine ward practice. The process indicators further suggest that the program required a manageable amount of instruction time, achieved reasonably consistent ward‐based patient instruction, and supported the maintenance of home exercise adherence, indicating acceptable implementation feasibility, continuity, and reproducibility. It may therefore represent a promising nurse‐led management option for supporting perioperative AVF functional establishment and preparing vascular access for clinical use. Future multicenter studies with stronger designs, longer follow‐up, and objective adherence monitoring are needed to confirm these findings and further evaluate long‐term AVF function and resource‐related outcomes.

## Author Contributions

Yang Tang and Mengya Wang contributed equally to this work and share first authorship and were responsible for conceptualization, methodology, literature review, intervention development, intervention implementation and management, formal analysis, and drafting of the manuscript; Mengyao Liu, Pinli Lin, and Xu Deng were responsible for study implementation and follow‐up; Jialu Xu, Mei Ling, and Yao Fang were responsible for data curation; Yuan Zhang and Yu Gao contributed to manuscript revision and content refinement; Fang Tang and Lili Deng acquired funding for the study; Yucheng Cao and Lili Deng were responsible for supervision, project advancement, and project administration.

## Funding

This work was supported by the General Program of the Basic and Applied Basic Research Foundation of Guangdong Province (Grant No. 2025A1515011049), the Guangdong Weiji Medical Development Foundation (Grant No. K‐202505‐1‐29), and the “Strengthening the Foundation” Project for Enhancing the Capacity of First‐level Disciplines in 2025 (Grant No. GZY2025GB0910).

## Disclosure

All authors read and approved the final manuscript.

## Ethics Statement

Ethical approval for this study was obtained from the Medical Ethics Committee of the Second Affiliated Hospital of Guangzhou University of Chinese Medicine (Approval No. ZF2024‐030‐01).

## Conflicts of Interest

The authors declare no conflicts of interest.

## Supporting Information

Additional supporting information can be found online in the Supporting Information section.

## Supporting information


**Supporting Information** The supporting materials include Supporting Tables S1–S7 and Supporting Figure S1, providing additional methodological details and supporting results.

## Data Availability

The data that support the findings of this study are available from the corresponding author upon reasonable request.
