# Supplementary material for: Development, Validation, and Evaluation of a Nurse‐Led Perioperative Staged Exercise Management Program for Arteriovenous Fistula: A Sequential Multiphase Study
Source: J Nurs Manag. 2026 Aug 9;2026:1430814. doi: 10.1155/jonm/1430814 (PMC13454739; doi:10.1155/jonm/1430814)
Supplement: Supplementary file 1 [file JONM-2026-1430814-s001.zip › Additional file 1 supplementary tables-revised-unmarked-final.docx]

**Supporting information**

**Development, Validation, and Evaluation of a Nurse-Led Perioperative Staged Exercise Management Program for Arteriovenous Fistula: A Sequential Multiphase Study**

Yang Tang^1†^, Mengya Wang^2†^, Mengyao Liu^1^, Pinli Lin^3^, Xu Deng^1^, Jialu Xu^1^, Mei Ling^1^, Yao Fang^1^, Yuan Zhang^1^, Yu Gao^4^, Fang Tang^5^, Yucheng Cao^1*^, Lili Deng^1*^

1: School of Nursing, Guangzhou University of Chinese Medicine, Guangzhou, Guangdong 510006, China

2: School of Nursing, Hunan University of Chinese Medicine, Changsha, Hunan 410208, China.

3: School of Nursing, Guangdong Pharmaceutical University, Guangzhou,Guangdong 510310, China.

4: Qionghai Institute of Nursing Excellence, Qionghai, Hainan 571434, China

5: The Second Affiliated Hospital of Guangzhou University of Chinese Medicine, Guangzhou, Guangdong 510120, China

**^†^These authors contributed equally as first authors.**

***These authors contributed equally as corresponding authors.**

**^*^Corresponding author:**
Yucheng Cao: jhudrph@outlook.com

Lili Deng: gzucmDll@163.com

**Supplementary Table captions:**

**Table S1. Detailed search strategies for each database**

#### **Table S2: Literature Characteristics**

Note: AVF, arteriovenous fistula; IG, intervention group; CG, control group; POD,postoperative day; RM, repetition maximum. Outcomes: ① vessel diameter; ② blood flow; ③ maturation rate/time; ④ adverse events or clinical usability.

**Table S3. Characteristics of Delphi experts**

**Table S4. Detailed operational procedures of the perioperative staged hand exercise program**

**Table S5. Schedule of assessments and data collection**

**Table S6. Results of Round 1 Delphi expert consultation**

Note:CV, coefficient of variation.

**Table S7. Within-group before and after changes and between-group comparisons of change scores for ultrasound outcomes after 8 weeks of staged exercise management**

Note: Δ is defined as the value at 8 weeks after intervention minus the preoperative baseline value.

Table S1. Detailed search strategies for each database

| Database | Search strategy |
| --- | --- |
| Pubmed | ((hemodialysis[tiab]) AND ((arteriovenous[tiab] AND fistula[tiab]) OR "arteriovenous fistula"[tiab] OR AVF[tiab]) AND (exercise[tiab] OR "exercise therapy"[tiab] OR "isometric exercise"[tiab] OR training[tiab] OR "upper limb exercise"[tiab] OR "arm exercise"[tiab])) |
| Embase | hemodialysis:ti,ab AND (arteriovenous:ti,ab AND fistula:ti,ab OR 'arteriovenous fistula':ti,ab OR avf:ti,ab) AND (exercise:ti,ab OR 'exercise therapy':ti,ab OR 'isometric exercise':ti,ab OR training:ti,ab OR 'upper limb exercise':ti,ab OR 'arm exercise':ti,ab) |
| EBSCO | ((TI hemodialysis OR AB hemodialysis) AND (((TI arteriovenous OR AB arteriovenous) AND (TI fistula OR AB fistula)) OR (TI "arteriovenous fistula" OR AB "arteriovenous fistula") OR (TI AVF OR AB AVF)) AND ((TI exercise OR AB exercise) OR (TI "exercise therapy" OR AB "exercise therapy") OR (TI "isometric exercise" OR AB "isometric exercise") OR (TI training OR AB training) OR (TI "upper limb exercise" OR AB "upper limb exercise") OR (TI "arm exercise" OR AB "arm exercise"))) |
| Cochrane Library | (hemodialysis:ti,ab,kw) AND ((arteriovenous:ti,ab,kw AND fistula:ti,ab,kw) OR "arteriovenous fistula":ti,ab,kw OR AVF:ti,ab,kw) AND (exercise:ti,ab,kw OR "exercise therapy":ti,ab,kw OR "isometric exercise":ti,ab,kw OR training:ti,ab,kw OR "upper limb exercise":ti,ab,kw OR "arm exercise":ti,ab,kw) |
| Web of Science | TS=(hemodialysis) AND (TS=(arteriovenous AND fistula) OR TS=("arteriovenous fistula") OR TS=(AVF)) AND (TS=(exercise) OR TS=("exercise therapy") OR TS=("isometric exercise") OR TS=(training) OR TS=("upper limb exercise") OR TS=("arm exercise")) |
| CNKI | ( (SU %= hemodialysis OR KY = hemodialysis) AND (SU %= arteriovenous fistula OR KY = arteriovenous fistula OR SU %= arteriovenous internal fistula OR KY = arteriovenous internal fistula OR SU %= arteriovenous * fistula OR KY = arteriovenous * fistula) AND (SU %= exercise OR KY = exercise OR SU %= exercise therapy OR KY = exercise therapy OR SU %= isometric exercise OR KY = isometric exercise OR SU %= training OR KY = training OR SU %= upper limb exercise OR KY = upper limb exercise OR SU %= arm exercise OR KY = arm exercise) ) |
| Wanfang | ((Title:hemodialysis OR Abstract:hemodialysis) AND (Title:(arteriovenous internal fistula OR arteriovenous fistula) OR Abstract:(arteriovenous internal fistula OR arteriovenous fistula)) AND (Title:(exercise OR exercise therapy OR isometric exercise OR training OR upper limb exercise OR arm exercise) OR Abstract:(exercise OR exercise therapy OR isometric exercise OR training OR upper limb exercise OR arm exercise))) |
| VIP | ( (T=hemodialysis OR K=hemodialysis) AND (T=arteriovenous fistula OR T=arteriovenous internal fistula OR K=arteriovenous fistula OR K=arteriovenous internal fistula) AND (T=exercise OR T=exercise therapy OR T=isometric exercise OR T=training OR T=upper limb exercise OR T=arm exercise OR K=exercise OR K=exercise therapy OR K=isometric exercise OR K=training OR K=upper limb exercise OR K=arm exercise) ) |
| CBM | (hemodialysis) AND (arteriovenous fistula OR arteriovenous internal fistula) AND (exercise OR exercise therapy OR isometric exercise OR training OR upper limb exercise OR arm exercise) |

#### Table S2: Literature Characteristics

| Author | Year | Country | N | | Intervention | Start time | Duration | Dose | Progression | Safety monitoring | Instruction/  follow-up | Adherence | Outcomes |
| --- | --- | --- | --- | --- | --- | --- | --- | --- | --- | --- | --- | --- | --- |
|  |  |  | IG | CG |  |  |  |  |  |  |  |  |  |
| Salimi[12] | 2013 | Iran | 25 | 25 | Isometric exercise and upper-arm tourniquet | POD3 | 2 weeks | 4 times/day;  5 × 5 s | Added ball squeezing, dumbbells,  and resistance band  by postoperative stage | Tourniquet pressure set so thrill disappeared while bruit remained audible | Pamphlet provided; early supervised training; then home practice | Not reported | ①②③④ |
| Kong[24] | 2014 | Korea | 10 | 8 | Handgrip exercise using a dynamometer (10RM) | Within  24 h if no abnormality | 4 weeks | Twice/day;  3 sets/session; 10RM intensity | 10RM recalculated weekly | Started only when no vascular dysfunction, thrombosis, swelling, or ischaemia was present | Guidance by research staff (no after discharge follow-up chain reported) | Not reported | ③④ |
| Fontsere[25] | 2016 | Spain | 38 | 31 | Elastic band resistance training for elbow, wrist, and hand | Within  24 h if no abnormality | 4 weeks | Twice/day | No explicit progression rule | Not clearly reported | Clinical and ultrasound assessment at 1 month by nurses and vascular access team | Not reported | ①②③④ |
| Chen[27] | 2020 | China | 60 | 58 | Audio visual “new fistula strengthening exercise” including finger grasping and wrist movements | POD 3 | 8 weeks | 4-5 times/day | Standardized video plus repeated practice; no explicit staged progression | Self-monitoring for weakened/disappeared thrill; nurse check at least once daily | Group practice; repeated video playback; nurses assessed mastery daily | Exercise log; family supervision; nurse feedback | ④ |
| Zhang [26] | 2020 | China | 44 | 43 | Five step fistula strengthening exercise | After wound healing and no bleeding/  infection | Until AVF maturation | Twice/day;  10 min/session | No explicit progression rule | Exercise should be tolerated by the patient | Not reported | Not reported | ④ |
| Tapia  Gonzalez[9] | 2021 | Spain | 30 | 30 | Protocolled isometric exercise program | Within  24 h if no abnormality | 8 weeks | Daily | Basic movements in weeks 1-4; added movements/load in weeks 4-8 | Adverse events and complications recorded | Program instruction provided; follow-up at weeks 4 and 8 | Monitoring sheet used | ①②③④ |
| Chen[10] | 2023 | Taiwan, China | 36 | 43 | Ball-squeezing with tourniquet compression | POD 2 | 12 weeks | 5 min/session; 2-6sessions/day | Compared exercise frequency and tourniquet compression | Adverse events recorded: bleeding, thrombosis, wound complications, infection | Individualised booklet; home-based exercise; clinicians assessed clinical usability | Not reported | ③④ |
| Tang [28] | 2023 | China | 45 | 45 | Finger, fist and tourniquet-assisted arm movements | After wound healing and no bleeding/  infection | 8 weeks | Early phase: 10 times/day;  later phase: 3 sets, 10 times/day | Staged progression from finger to fist to tourniquet assisted exercise | Tourniquet tightness set so one finger could be inserted and blood flow was not interrupted; wound and bleeding monitored | Demonstration video and nurse instruction | Not reported | ③④ |

Note: AVF, arteriovenous fistula; IG, intervention group; CG, control group; POD, postoperative day; RM, repetition maximum.

Outcomes: ① vessel diameter; ② blood flow; ③ maturation rate/time; ④ adverse events or clinical usability.

Table S3. Characteristics of Delphi experts

| **Characteristics** | **Round1** | **Round2** |
| --- | --- | --- |
| Number | 15 | 14 |
| Sex |  |  |
| Male | 7 (46.7%) | 6 (42.9%) |
| Female | 8 (53.3%) | 8 (57.1%) |
| Educational background |  |  |
| Bachelor’s degree | 4 (26.7%) | 4 (28.6%) |
| Master’s degree | 7 (46.7%) | 6 (42.9%) |
| Doctoral degree | 4 (26.7%) | 4 (28.6%) |
| Profession titles |  |  |
| Senior | 5 (33.3%) | 5 (35.7%) |
| Associate senior | 7 (46.7%) | 6 (42.9%) |
| Mid-level | 3 (20.0%) | 3 (21.4%) |
| Professional experience (years) |  |  |
| 10-20 | 8 (53.3%) | 7 (50.0%) |
| 20-30 | 7 (46.7%) | 7 (50.0%) |
| Mentor type |  |  |
| Master supervisor | 6 (40.0%) | 6 (42.9%) |
| PhD supervisor | 2 (13.3%) | 2 (14.3%) |
| Clinical Expert | 7 (46.7%) | 6 (42.9%) |
| Professional Field |  |  |
| Nephrology | 6 (40.0%) | 6 (42.9%) |
| Hemodialysis Care | 5 (33.3%) | 5 (35.7%) |
| Vascular Surgery | 3 (20.0%) | 2 (14.3%) |
| Rehabilitation Medicine | 1 (6.7%) | 1 (7.1%) |

Table S4. Detailed operational procedures of
 the perioperative staged hand exercise program

| **Stage** | **Timing** | **Detailed operational procedures** |
| --- | --- | --- |
| Stage 1 | First 12–24 hours after surgery | Patients were instructed to adopt a supine or semi-recumbent position, with the operated upper limb slightly elevated on a soft or wedge pillow so that the elbow to wrist line was approximately 15° above heart level to promote venous return and reduce swelling. The elbow was kept slightly flexed and the wrist in a neutral position to avoid pressure on the anastomotic area. Elevation during the daytime was encouraged whenever possible, and the temperature of the dorsum of the hand and capillary refill were checked every 2 to 3 hours. Intermittent gentle fingertip activity was allowed to prevent stiffness. |
| Stage 2 | Postoperative day 2 | Patients were positioned sitting or semi-reclining, with relaxed shoulders, elbows flexed at 30° to 45°, and the forearm supported on a table or pillow. Fingertip exercise consisted of lightly tapping the supporting surface as if playing the piano, and opposition exercise consisted of touching the thumb to each of the other fingers in sequence. Each exercise lasted about 3 minutes and was performed once in the morning and once in the evening. Intensity was limited to a level at which patients could speak comfortably without holding their breath, and large-amplitude wrist movements or excessive force that could cause traction discomfort were avoided. |
| Stage 3 | Postoperative days 3–6 | Wrist exercise followed the sequence of extension, flexion, radial deviation, ulnar deviation, pronation, and supination, with a moderate range of motion and even rhythm, holding each movement for about 5 seconds and cycling for approximately 3 minutes. Fist clenching consisted of alternating empty fist clenching with full finger extension. If tolerated, suspended wrist fist clenching, with light forearm suspension and the wrist kept neutral, was added. Each exercise lasted about 3 minutes and was performed twice daily. |
| Stage 4 | Postoperative days 7–14 | Ball squeezing used a soft elastic ball and followed a cycle of squeezing and holding for about 5 seconds, then fully extending the hand and relaxing for 5 seconds, for approximately 3 minutes. During elbow flexion ball squeezing, the elbow was slowly flexed to about 90° during the holding phase, briefly maintained, and then returned to the starting position before the hand was relaxed for 5 seconds. This cycle also lasted about 3 minutes and was performed twice daily. If soreness or fatigue developed, the holding time could be reduced from 5 seconds to 3 seconds and gradually increased again after tolerance improved. |
| Stage 5 | Postoperative day 14 to week 8 | Each full session followed the sequence of warm-up, core training, and relaxation. Warm-up lasted 1 to 2 minutes and consisted of light wrist and finger movements. The core component included fingertip activity, opposition exercise, wrist exercise, fist clenching, ball squeezing, and elbow flexion ball squeezing, with each module lasting about 3 minutes. Relaxation lasted 2 to 3 minutes and involved full finger extension, light shaking, and, if necessary, local warm compresses. Frequency remained twice daily, with a total session duration of 15 to 20 minutes. During weeks 3 and 4, the duration of each module remained unchanged while the ball-squeezing hold was increased from 5 seconds to 6 to 7 seconds if tolerated. During weeks 5 to 8, repetitions were increased by about 10% to 20% if tolerated, or a second round of the core module was added to either the morning or evening session, with the total duration not exceeding 25 minutes. |

Table S5. Schedule of assessments and data collection

| **Item** | **Pre-intervention** | **8 weeks after intervention** | **12 weeks after intervention** |
| --- | --- | --- | --- |
| Baseline characteristics | √ |  |  |
| Surgery-related information | √ |  |  |
| RA PSV | √ |  |  |
| UA diameter | √ |  |  |
| UA PSV | √ |  |  |
| BA diameter | √ | √ |  |
| BA blood flow | √ | √ |  |
| RA diameter | √ | √ |  |
| DV diameter | √ | √ |  |
| Occurrence of complications |  | √ | √ |
| AVF maturation status |  | √ | √ |
| Time to maturation | Continuously recorded during follow-up until maturation | | |

Table S6. Results of Round 1 Delphi expert consultation

| **Item** | **Importance score, mean ± SD** | **CV** |
| --- | --- | --- |
| I. Patient eligibility and initial assessment | 4.60±0.51 | 0.11 |
| 1.1 Surgical and fistula assessment | 4.87±0.35 | 0.07 |
| 1.1.1 Confirm that the patient meets the starting time for exercise at this stage | 4.87±0.35 | 0.07 |
| 1.1.2 No active bleeding or obvious hematoma at the fistula anastomosis | 4.93±0.26 | 0.05 |
| 1.1.3 Slight thrill can be palpated or a faint bruit can be heard over the fistula vessel | 4.73±0.46 | 0.10 |
| 1.2 Assessment of baseline patient status | 4.33±0.62 | 0.14 |
| 1.2.1 The patient is conscious and able to understand and follow instructions | 4.60±0.63 | 0.14 |
| 1.2.2 Normal skin temperature, color and sensation of the operated hand, with no severe edema | 4.53±0.64 | 0.14 |
| 1.2.3 Assess whether the patient has any pre-existing limb dysfunction | 3.80±0.86 | 0.23 |
| 1.3 Assessment of patient willingness and support system | 4.20±0.68 | 0.16 |
| 1.3.1 Patient willingness to participate in exercise | 4.40±0.63 | 0.14 |
| 1.3.2 Feasibility of family supervision and assistance | 4.00±0.76 | 0.19 |
| II. Implementation of the staged exercise program | 4.40±0.51 | 0.12 |
| 2.1 Postoperative 12-24 hours (positioning phase) | 4.40±0.63 | 0.14 |
| 2.1.1 Position the patient supine or semi-recumbent with the operated upper limb elevated on a soft pillow | 4.67±0.49 | 0.10 |
| 2.1.2 Ensure slight elbow flexion and a neutral wrist position to avoid pressure on the anastomosis | 4.60±0.51 | 0.11 |
| 2.1.3 Teach the patient and family to self-check the temperature of the hand dorsum and capillary refill every 2-3 hours | 3.93±0.80 | 0.20 |
| 2.2 Postoperative day 2 (fingertip activity phase) | 4.33±0.62 | 0.14 |
| 2.2.1 Instruct the patient to sit with relaxed shoulders, elbows flexed 30-45°, and the forearm supported | 4.33±0.72 | 0.17 |
| 2.2.2 Teach piano-like fingertip tapping on the tabletop in sequence | 4.40±0.63 | 0.14 |
| 2.2.3 Teach thumb-to-finger opposition with each of the other four fingers in turn | 4.40±0.63 | 0.14 |
| 2.2.4 Ensure an exercise frequency of once each morning and evening with an interval of ≥8 hours | 4.27±0.70 | 0.16 |
| 2.3 Postoperative days 3-6 (wrist and fist clenching phase) | 4.27±0.70 | 0.16 |
| 2.3.1 Add the wrist sequence of extension → flexion → radial deviation → ulnar deviation → pronation → supination | 4.33±0.72 | 0.17 |
| 2.3.2 Teach alternating empty-fist clenching and full hand opening | 4.33±0.72 | 0.17 |
| 2.3.3 Emphasise a moderate range of motion and even rhythm, avoiding sustained forceful gripping for >5 seconds | 4.20±0.68 | 0.16 |
| 2.4 Postoperative days 7-14 (ball squeezing strengthening phase) | 4.40±0.63 | 0.14 |
| 2.4.1 Introduce a soft elastic ball and teach the cycle “squeeze and hold for 5 seconds → fully open and relax for 5 seconds” | 4.53±0.64 | 0.14 |
| 2.4.2 Teach the elbow flexion ball squeezing movement | 4.47±0.64 | 0.14 |
| 2.4.3 Guide the patient to adjust the holding time according to soreness or tightness | 4.20±0.68 | 0.16 |
| 2.5 Postoperative day 14 to week 8 (consolidation and progression phase) | 4.27±0.70 | 0.16 |
| 2.5.1 Establish a complete “warm-up - core - relaxation” sequence | 4.27±0.70 | 0.16 |
| 2.5.2 During weeks 3-4, guide the patient to increase the ball-squeeze hold from 5 seconds to 6-7 seconds | 4.33±0.72 | 0.17 |
| 2.5.3 During weeks 5-8, guide the patient to increase repetitions by 10%-20% | 4.20±0.68 | 0.16 |
| III. Monitoring and adjustment during exercise | 4.47±0.52 | 0.12 |
| 3.1 Monitoring of intensity and tolerance | 4.13±0.74 | 0.18 |
| 3.1.1 Supervise throughout to ensure intensity remains at a level where the patient can speak comfortably and does not hold their breath | 4.60±0.51 | 0.11 |
| 3.1.2 Record soreness, numbness, tightness, pain and other responses after each exercise session | 3.67±0.90 | 0.25 |
| 3.2 Monitoring of fistula status and complications | 4.80±0.41 | 0.09 |
| 3.2.1 Assess whether fistula thrill and bruit remain satisfactory before each exercise session | 4.80±0.41 | 0.09 |
| 3.2.2 Monitor and record complications such as arm swelling, subcutaneous hematoma and anastomotic bleeding | 4.73±0.46 | 0.10 |
| 3.2.3 Stop immediately and inform the physician if abnormalities occur, such as weakening or disappearance of thrill or bruit | 4.80±0.41 | 0.09 |
| 3.3 Adherence monitoring | 4.20±0.68 | 0.16 |
| 3.3.1 Instruct the patient or family to complete the Hand Exercise Record Card | 4.20±0.68 | 0.16 |
| IV. Health education and management | 4.33±0.49 | 0.11 |
| 4.1 Knowledge education | 4.20±0.68 | 0.16 |
| 4.1.1 Explain the purpose of exercise at each stage to the patient | 4.27±0.70 | 0.16 |
| 4.1.2 Clearly inform the patient of the overall program duration and the timing of each stage | 4.13±0.74 | 0.18 |
| 4.2 Behavioral guidance | 4.47±0.52 | 0.12 |
| 4.2.1 Emphasise the importance of adherence to the prescribed exercise frequency and duration | 4.20±0.68 | 0.16 |
| 4.2.2 Teach the patient to recognise and report “red-flag” signs | 4.73±0.46 | 0.10 |
| V. Nurses’ management of patients | 4.20±0.56 | 0.13 |
| 5.1 Implementation and recording | 4.20±0.68 | 0.16 |
| 5.1.1 On the first day of each stage, nurses provide standardized teaching and assessment | 4.47±0.64 | 0.14 |
| 5.1.2 Nurses regularly review and collect the Exercise Record Card to assess adherence | 3.93±0.80 | 0.20 |
| 5.2 Follow-up and handover | 4.20±0.56 | 0.13 |
| 5.2.1 Establish a after discharge follow-up mechanism to monitor home exercise performance | 4.60±0.51 | 0.11 |
| 5.2.2 Communicate exercise performance to physicians as part of fistula assessment | 3.87±0.83 | 0.22 |

Note:CV, coefficient of variation.

Table S7. Within-group before and after changes and between-group comparisons of change scores for ultrasound outcomes after 8 weeks of staged exercise management

| Outcome  (postoperative - preoperative) | **Control group** | | **Intervention group** | | **Between-group comparison of Δ** | |
| --- | --- | --- | --- | --- | --- | --- |
|  | **Z** | ***P*** | **Z** | ***P*** | **Z** | ***P*** |
| BA flow | -5.579 | <0.001 | -5.579 | <0.001 | -3.696 | ＜0.001 |
| BA diameter | -5.582 | <0.001 | -5.580 | <0.001 | -2.850 | 0.004 |
| RA diameter | -5.581 | <0.001 | -5.581 | <0.001 | -1.917 | 0.055 |
| DV diameter | -5.581 | <0.001 | -5.580 | <0.001 | -0.506 | 0.613 |

Note: Δ is defined as the value at 8 weeks after intervention minus the preoperative baseline value.
