# Supplementary material for: Development, Validation, and Evaluation of a Nurse‐Led Perioperative Staged Exercise Management Program for Arteriovenous Fistula: A Sequential Multiphase Study
Source: J Nurs Manag. 2026 Aug 9;2026:1430814. doi: 10.1155/jonm/1430814 (PMC13454739; doi:10.1155/jonm/1430814)
Supplement: Supplementary file 1 [file JONM-2026-1430814-s001.zip › Additional file 2 supplementary figures-revised-unmarked-final.docx]

**Supporting information**

**Development, Validation, and Evaluation of a Nurse-Led Perioperative Staged Exercise Management Program for Arteriovenous Fistula: A Sequential Multiphase Study**

Yang Tang^1†^, Mengya Wang^2†^, Mengyao Liu^1^, Pinli Lin^3^, Xu Deng^1^, Jialu Xu^1^, Mei Ling^1^, Yao Fang^1^, Yuan Zhang^1^, Yu Gao^4^, Fang Tang^5^, Yucheng Cao^1*^, Lili Deng^1*^

1: School of Nursing, Guangzhou University of Chinese Medicine, Guangzhou, Guangdong 510006, China

2: School of Nursing, Hunan University of Chinese Medicine, Changsha, Hunan 410208, China.

3: School of Nursing, Guangdong Pharmaceutical University, Guangzhou,Guangdong 510310, China.

4: Qionghai Institute of Nursing Excellence, Qionghai, Hainan 571434, China

5: The Second Affiliated Hospital of Guangzhou University of Chinese Medicine, Guangzhou, Guangdong 510120, China

**^†^These authors contributed equally as first authors.**

***These authors contributed equally as corresponding authors.**

**^*^Corresponding author:**
Yucheng Cao: jhudrph@outlook.com

Lili Deng: gzucmDll@163.com

**Supplementary Figure captions:**

**Figure S1. Flow diagram of the literature screening process for studies on hand exercise and arteriovenous fistula maturation in patients with end-stage renal disease.**

Figure S1. Flow diagram of the literature screening process for studies on hand exercise and arteriovenous fistula maturation in patients with end-stage renal disease.
