# Supplementary material for: Development, Validation, and Evaluation of a Nurse‐Led Perioperative Staged Exercise Management Program for Arteriovenous Fistula: A Sequential Multiphase Study
Source: J Nurs Manag. 2026 Aug 9;2026:1430814. doi: 10.1155/jonm/1430814 (PMC13454739; doi:10.1155/jonm/1430814)
Supplement: Supplementary file 1 [file JONM-2026-1430814-s001.zip › Figure S1-final.pdf]

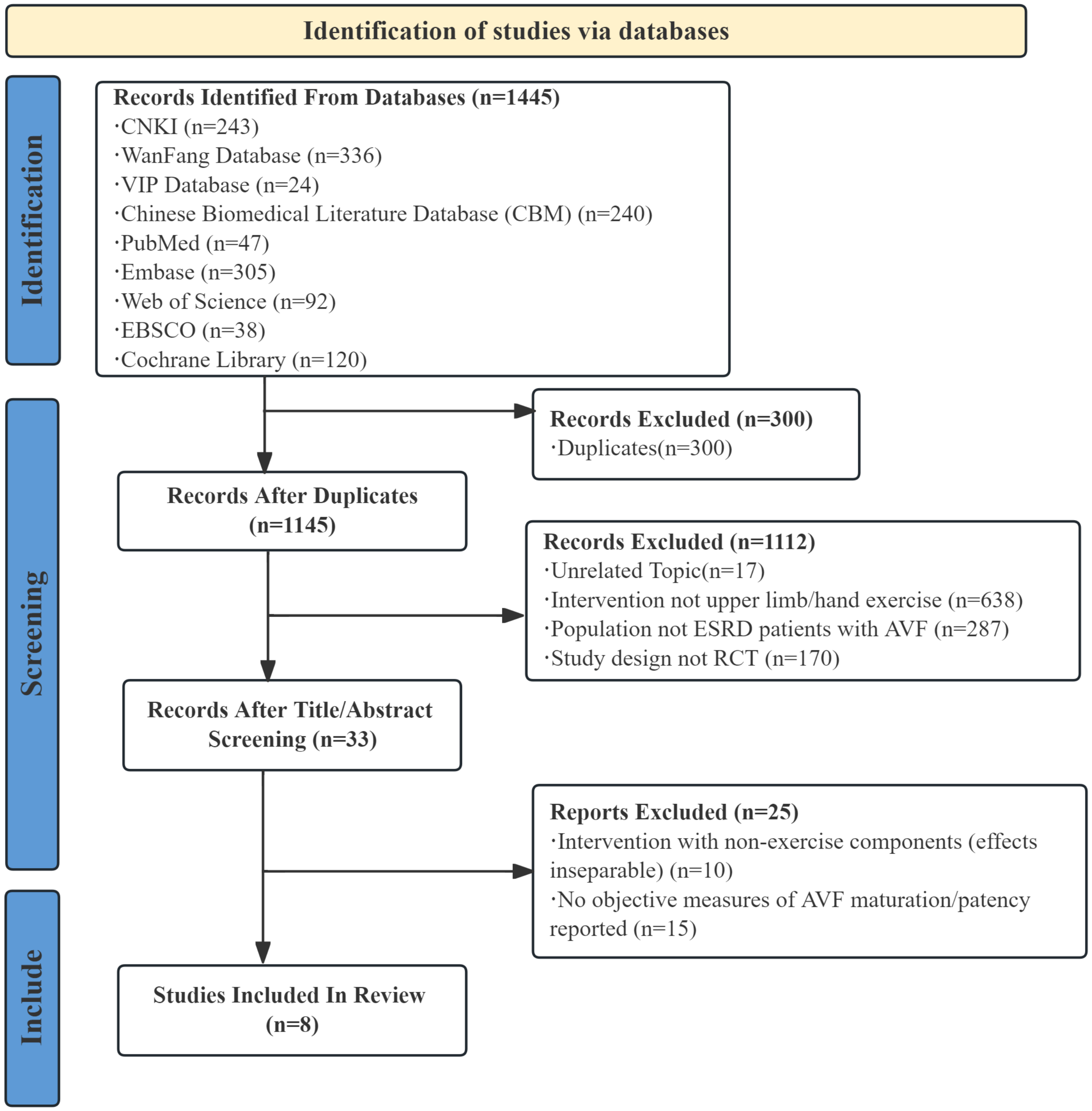

Identification of studies via databases
Identification
Records Identified From Databases (n=1445)
·CNKI (n=243)
·WanFang Database (n=336)
·VIP Database (n=24)
·Chinese Biomedical Literature Database (CBM) (n=240)
·PubMed (n=47)
·Embase (n=305)
·Web of Science (n=92)
·EBSCO (n=38)
·Cochrane Library (n=120)
Screening
Records Excluded (n=300)
·Duplicates(n=300)
Records After Duplicates (n=1145)
Records Excluded (n=1112)
·Unrelated Topic(n=17)
·Intervention not upper limb/hand exercise (n=638)
·Population not ESRD patients with AVF (n=287)
·Study design not RCT (n=170)
Records After Title/Abstract Screening (n=33)
Reports Excluded (n=25)
·Intervention with non-exercise components (effects inseparable) (n=10)
·No objective measures of AVF maturation/patency reported (n=15)
Include
Studies Included In Review (n=8)
